# Chinese herbal medicine, Tongxieyaofang, alleviates diarrhea via gut microbiota remodeling: evidence from network pharmacology and full-length 16S rRNA gene sequencing

**DOI:** 10.3389/fcimb.2024.1502373

**Published:** 2024-11-15

**Authors:** Haoqing Shao, Liping Wang, Hualing Zhang

**Affiliations:** ^1^ School of Traditional Chinese Medicine, Hunan University of Medicine, Huaihua, Hunan, China; ^2^ Research and Development Center, Kangpu Pharmaceutical Co., Ltd., Changde, Hunan, China

**Keywords:** Chinese medicine, intestinal mucosal microbiota, diarrhea, Tongxieyaofang (TXYF), full-length 16S rRNA gene sequencing

## Abstract

**Background:**

Tongxieyaofang (TXYF) was a traditional Chinese medicine (TCM) formula for the treatment of diarrhea with liver stagnation and spleen deficiency syndrome, but the potential targets and mechanisms have not been fully clarified. This study aims to explore the potential mechanisms of TXYF in alleviating diarrhea using network pharmacology and full-length 16S rRNA gene sequencing.

**Methods:**

Network pharmacology was applied to identify bioactive compounds and potential targets involved in the role of TXYF in alleviating diarrhea. Meanwhile, a model of diarrhea with liver stagnation and spleen deficiency syndrome was constructed by intragastric administration of *Folium senna* extract combined with restraint and tail pinch stress. The effect of TXYF on intestinal mucosal microbiota of diarrhea mice was analyzed by full-length 16S rRNA gene sequencing.

**Results:**

Network pharmacology analysis showed that kaempferol, wogonin, naringenin, and nobiletin were compounds associated with the efficacy of TXYF. TXYF may alleviate diarrhea via multiple BPs and pathways, including TNF signaling pathway, IL-17 signaling pathway, and Toll-like receptor signaling pathway, which are involved in TCM-gut microbiota-host interactions. Then, we found that TXYF administration reshaped the diversity and composition of the intestinal mucosal microbial community of diarrhea mice. *Lactobacillus*, primarily *Lactobacillus johnsonii*, was enriched by the administration of TXYF. After TXYF administration, the abundance of *Lactobacillus*, particularly *Lactobacillus johnsonii*, was enriched.

**Conclusion:**

Oral administration of TXYF may alleviate diarrhea through remodeling intestinal mucosal microbiota. Promoting the colonization of beneficial commensal bacteria in the intestinal mucosa through gut microbiota-host interactions may be a potential mechanism of TXYF in the treatment of diarrhea.

## 1 Introduction

Diarrhea is one of the most common symptoms in digestive. The incidence of diarrhea ranges from 2.2% to 73.8%, with variability depending on the definition applied (Dionne and Mbuagbaw, 2023). Diarrhea remains a common issue in clinical practice, and finding effective administration strategies is still necessary. Tongxieyaofang (TXYF), a traditional Chinese medicine (TCM) formula, consists of Rhizoma *Atractylodis Macrocephalae* (BaiZhu, dried rootstocks of *Atractylodes macrocephala* Koidz.), Radix *Paeoniae Alba* (BaiShao, dried roots of *Paeonia lactiflora* Pall.), Pericarpium *Citri Reticulatae* (ChenPi, dried pericarps of *Citrus reticulata* Blanco and its Cultivars), and Radix *Saposhnikoviae* (FangFeng, dried roots of *Saposhnikovia divaricata* (Turcz.) Schischk.) in a ratio of 3:2:1.5:1. TXYF, initially recorded in “Danxi’s Experiential Therapy” during the 14th century, was a treatment with proven effects for diarrhea accompanied by abdominal pain resulting from liver stagnation and spleen deficiency. It was officially recommended as a fundamental prescription for patients afflicted with diarrhea associated with liver stagnation and spleen deficiency syndrome in the Expert Consensus on the TCM Diagnosis and Treatment for Diarrhea (2017). According to TCM theory, TXYF can soothe the liver and regulate Qi, strengthen the spleen, transform dampness, and stop diarrhea. Randomized controlled clinical studies have shown that TXYF, as a primary prescription, has significant efficacy in relieving loose stools, abdominal discomfort, and bloating in IBS-D (Tang et al., 2019; Liang et al., 2022), which provides clinical evidence for its effect on alleviating diarrhea and abdominal pain. Although the clinical efficacy is definite, the potential mechanism of TXYF in stopping diarrhea needs to be further clarified.

Alterations in gut microbiota, also called dysbacteriosis, have been found in many gastrointestinal diseases with diarrhea as the main clinical manifestation (Qiao et al., 2023; Wu et al., 2023; Zhou et al., 2023; Luo et al., 2024). In previous work, we also observed significant differences in the intestinal cavity and intestinal mucosal microbiota between mice with diarrhea due to liver stagnation and spleen deficiency and control mice (Yuan et al., 2020; Zhang et al., 2020). Fecal microbiota transplantation of human fecal samples into germ-free mice can also construct human disease phenotype models (Wang et al., 2022). Targeted manipulation of gut microbiota to treat diarrhea also has shown great potential and attracted wide attention (Qiao et al., 2024; Zhou et al., 2024). Under this background, researchers specializing in TCM uncovered that TCM can modulate the structure and metabolism of the gut microbiota to exert therapeutic effects (Shao et al., 2020; Zhu et al., 2023; Wu et al., 2024). These discoveries provide a new scientific rationale for the application of TCM in modern society, particularly in gastrointestinal health and disease management. Delving into the effects of TCM on the gut microbiota can help reveal the modern scientific basis for its traditional efficacy and pave the way for the development of novel treatment approaches. Therefore, we designed to investigate if the effect of TXYF in alleviating diarrhea with liver stagnation and spleen deficiency syndrome is associated with gut microbiota regulation.

Network pharmacology, a novel model characterized by ‘multi-component and network target’ approaches in TCM research, has been introduced as an encouraging method to study the mechanisms of herbal formulas (Yao et al., 2022; Zhao et al., 2023). In this study, we aimed to identify the potential targets of TXYF alleviating diarrhea through network pharmacology and assess the effects of TXYF on the intestinal mucosa microbiota in mice with diarrhea with liver stagnation and spleen deficiency syndrome using full-length 16S rRNA gene sequencing.

## 2 Materials and methods

### 2.1 Animals and ethics statement

Fifteen one-month-old male specific-pathogen-free Kunming mice, weighing (20 ± 2) g, were purchased from the Hunan SJA Laboratory Animal Co., Ltd (License No: SCXK (Xiang) 2016-0002). Mice were housed in the barrier environment of the Center for Experimental Animals of the Hunan University of Chinese Medicine with (23-25) °C, (47-53) % humidity, 12h light/dark cycle, with free access to water and food during adaptive feeding. Animal experiments were performed following the National Guidelines for Experimental Animal Welfare, approved by the Animal Ethics and Welfare Committee of Hunan University of Chinese Medicine.

### 2.2 Preparation of experimental decoctions

#### 2.2.1 *Folium senna* extract

All traditional Chinese herbs were purchased from the pharmacy of the First Affiliated Hospital of Hunan University of Chinese Medicine. The authenticated voucher specimens were deposited at School of Chinese Medicine, Hunan University of Chinese Medicine. *Folium senna* (Fanxieye, dried leaf of *Cassia angustifolia* Vahl or *Cassia acutifolia* Delile) samples (500 g) were cut into small pieces and soaked in boiled water (5000 mL) for 10 min. Then, the filtrates were evaporated to 500 mL with a thermostat water bath at 75°C to get the *Folium senna* extract with a crude drug concentration of 1 g/mL, which was stored at 4°C for model preparation later.

#### 2.2.2 TXYF decoction

The TXYF herbal materials (45 g), consisting of Rhizoma *Atractylodis Macrocephalae* (18 g), Radix *Paeoniae Alba* (12 g), Pericarpium *Citri Reticulatae* (9 g), and Radix *Saposhnikoviae* (6 *g*), were soaked in cold water (400 mL) for 30 minutes before boiled for 30 minutes. The filtrates were collected, and the herb residues were decocted again in the same way. Two times’ filtrates were mixed and evaporated to 100 mL to get the TXYF decoction with a crude drug concentration of 0.24 g/mL, which was stored at 4°C for drug administration later.

### 2.3 Process of animal experiment

After three days of adaptive feeding in a barrier environment, mice were randomly divided into a control group (gtcm), a placebo group (gtmm), and a TXYF group (gttm), with 5 mice in each group. The repeated stress-related diarrhea model was established referring to our previous study (Yuan et al., 2020; Zhang et al., 2020). Briefly, mice in the placebo and TXYF groups were gavage with *Folium Senna* extract at a dose of 17.5 mL/kg/d after fasting for 12 hours. At the period of *Folium Senna* administration, each mouse in the placebo and TXYF groups was put in a 50 mL centrifuge tube separately to restrict its activities for 1 hour/day, with its tail nipped at the distal 1/3 using a binder clip at the same time. Mice in the control group were given the same dose of sterile water and free access to water, food, and activities during the period of modeling. After seven days of continuous modeling, mice in the placebo and TXYF groups were successfully induced symptoms of diarrhea with liver stagnation and spleen deficiency syndrome. Then, mice in the TXYF group were gavage with 0.35 mL TXYF decoction twice a day for three days. The control and placebo groups were given the same dose of sterile water at the same time.

At the end of the TXYF treatment period, mice were sacrificed by cervical dislocation. The small intestinal mucosa samples of mice were collected referring to our previous study (Shao et al., 2020; Zhang et al., 2020). Briefly, the abdominal cavity of the mouse was opened at an ultra-clean workbench to take out the small intestine. To sample the mucosa conveniently, the intestinal wall was cut along the long axis. A sterile cover glass was used to scrape the intestinal mucosa tissue. All samples were collected and marked separately and stored at -80°C for further PacBio SMRT sequencing.

### 2.4 Network pharmacology analysis

All ingredients in the four Chinese herbal medicines of TXYF were retrieved from the traditional Chinese medicine systems pharmacology (TCMSP) database (Ru et al., 2014) (https://tcmspw.com/tcmsp.php). Drug-likeness (DL) and oral bioavailability (OB) were two crucial properties of absorption, distribution, metabolism, and excretion (ADME)-related parameters of ingredients. To obtain the ideal potential bioactive compounds, we set the screening criteria of DL greater than or equal to 0.18 and OB greater than or equal to 30%. Protein targets related to every active ingredient of TXYF were obtained using TCMSP databases and converted to target names in *Homo sapiens* using UniProtKB (UniProt, 2021) (https://www.uniprot.org/).

Human genes associated with diarrhea were identified from the GeneCards database (Safran et al., 2010) (https://www.genecards.org) with the keyword “diarrhea”. Then, the potential targets of TXYF in treating diarrhea were obtained by a comprehensive analysis of the direct related targets of TXYF and the diarrhea targets using the online tool Venny2.1 (https://bioinfogp.cnb.csic.es/tools/venny/index.html).

Potential targets for TXYF treat diarrhea were analyzed using the online database STRING11.0 (https://string-db.org/) to construct a functional protein-protein interaction (PPI) network of multiple proteins (Szklarczyk et al., 2019). To obtain appropriate confidence in identified protein interactions in the PPI network, we set the screening criteria of interaction scores greater than 0.9. To evaluate the role of each target in the PPI network, hub targets were identified using four different analysis methods (Degree, Closeness, MCC, and EcCentricity) in “cytoHubba” of cytoscape3.8.0 software (Shannon et al., 2003). Then, active compounds related to hub targets were screened and visualized with a Sankey diagram to introduce the relationship between hub targets and corresponding bioactive ingredients.

The “clusterProfiler” package in R software (v3.6.3) was employed to conduct GO and KEGG enrichment analysis.

### 2.5 Full-length 16S rRNA gene sequencing on the PacBio SMRT platform

Total bacterial genomic DNA was extracted from intestinal mucosa samples using the E.Z.N.A.^®^ Bacterial DNA Kit (OMEGA, USA). The quality and quantity of the extracted genomic DNA were checked using a 0.8% agarose gel electrophoresis and an ultraviolet spectrophotometer.

For amplification of full-length 16S rRNA gene, the following primers were used: forward primer 27F (5’-AGAGTTTGATCMTGGMCTCAG-3’) and reverse primer 1492R (5’-ACCTTGTTACGACTT-3’). Polymerase chain reaction (PCR) amplification of 16S rRNA genes was conducted using the Q5^®^ High-Fidelity DNA Polymerase (New England BioLabs, USA). Amplified DNA products were checked using a 2% agarose gel electrophoresis and purified using Axygen^®^ AxyPrep DNA Gel Extraction Kit. The quantity of amplified DNA products was determined using Quant-iT PicoGreen dsDNA Assay Kit. Mock community PacBio library was constructed using PacBio SMRTbell Template Prep Kit 1.0. The prepared DNA library was sequenced on the PacBio Sequel platform by Shanghai Personal Biotechnology Co., Ltd.

### 2.6 Bioinformatics analysis

Quality control of raw sequencing reads was processed to generate high-quality sequences using quantitative insights into microbial ecology (QIIME, v1.8.0) software. The high-quality sequences were clustered into operational taxonomic units (OTUs) at a more than 97% similarity threshold by UCLUST. OTU taxonomic classification was carried out by BLAST searching the representative sequence of each OTU set against the Greengenes database. Bioinformatics analysis was performed based on a filtered OTUs table, in which OTUs containing less than 0.001% of total sequences across all samples were discarded. Alpha and beta diversity were introduced to distinguish the diversity within samples and between samples. For alpha diversity analysis, observed species, Chao1, and Shannon indexes were calculated in the QIIME program and visualized by GraphPad Prism 8. For beta diversity analysis, nonmetric multidimensional scaling (NMDS) analysis was performed based on unweighted and weighted UniFrac distances using R. The analysis of similarities (ANOSIM) based on unweighted and weighted UniFrac distances was carried out to compare different groups by QIIME. Linear discriminant analysis (LDA) effect size (LEfSe) (Segata et al., 2011) was conducted to define microbial biomarkers of the three groups, which provided biological class explanations to establish statistical significance, biological consistency, and effect size estimation of predicted biomarkers. The threshold on the logarithmic LDA score for distinguishing features was set to 4.0. Predictive functional profiling of microbial communities was conducted using 16S rRNA data by Phylogenetic Investigation of Communities by Reconstruction of Unobserved States (PICRUSt) (Langille et al., 2013) in the context of the Kyoto encyclopedia of genes and genomes (KEGG). The raw data will be made available by the authors, without undue reservation, to any qualified researcher.

### 2.7 Statistical analysis

The data were expressed as mean ± standard deviation (SD) where applicable. SPSS 22.0 and GraphPad Prism 8.0 were used for statistical analysis. Because the data does not conform to the normal distribution, the Kruskal-Wallis H test and Mann-Whitney U test were used to evaluate the statistical significance of the differences between the three groups and the two groups respectively. Differences were considered statistically significant when *p* < 0.05. The threshold of *p*-value was determined with the original false discovery rate (FDR) method of Benjamini and Hochberg (BH) in multiple comparisons.

## 3 Results

### 3.1 Network pharmacology analysis

#### 3.1.1 Potential targets for TXYF treat diarrhea

**Figure 1:**
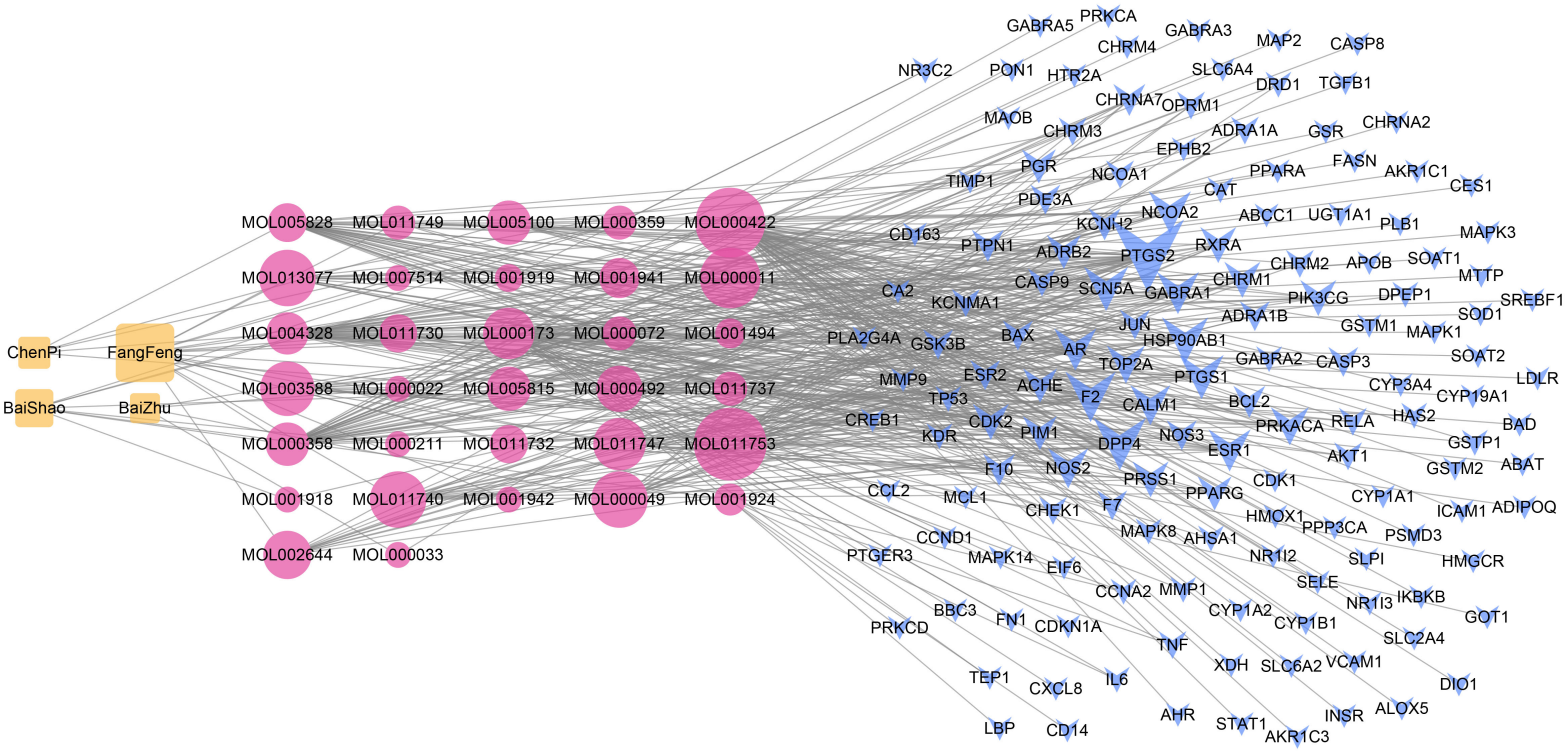
The herb-compound-target (CT) network. The orange nodes stand for herbs in TXYF. The red nodes stand for bioactive compounds in TXYF. The blue nodes stand for targets responding to the efficacy of TXYF. The grey lines stand for corresponding connections between different category nodes. The size of each node indicates the number of connections. The larger the node, the more the connections.

After filtered by the ADME threshold (OB ≥ 30% and DL ≥ 0.18) and removed the duplicates, a total of 32 bioactive ingredients in TXYF were screened by the TCMSP database (**Supplementary Table 1**). Based on identified bioactive compounds in TXYF, a total of 145 potential target genes responding to compounds of TXYF were retrieved using the TCMSP data server. An herb-compound-target (CT) network was constructed by Cytoscape 3.8.0 software, which included 181 nodes (four herb nodes, 32 bioactive compound nodes, and 145 compound-associated target nodes) and 431 compound-target corresponding connections (**Figure 1**). According to the degree of the nodes in the CT network, kaempferol (degree = 62), wogonin (degree = 46), beta-sitosterol (degree = 39), naringenin (degree = 38), nobiletin (degree = 35), 5-O-Methylvisamminol (degree = 25) were recognized as main compounds of TXYF.

Targets related to diarrhea were obtained from the GeneCards database. Finally, 2 698 diarrhea-related targets (relevance score > median) were incorporated into the study. There were 94 intersection targets between targets responding to compounds of TXYF and targets related to diarrhea, which were recognized as potential targets for TXYF treat diarrhea.

#### 3.1.2 GO and KEGG enrichment analysis

**Figure 2:**
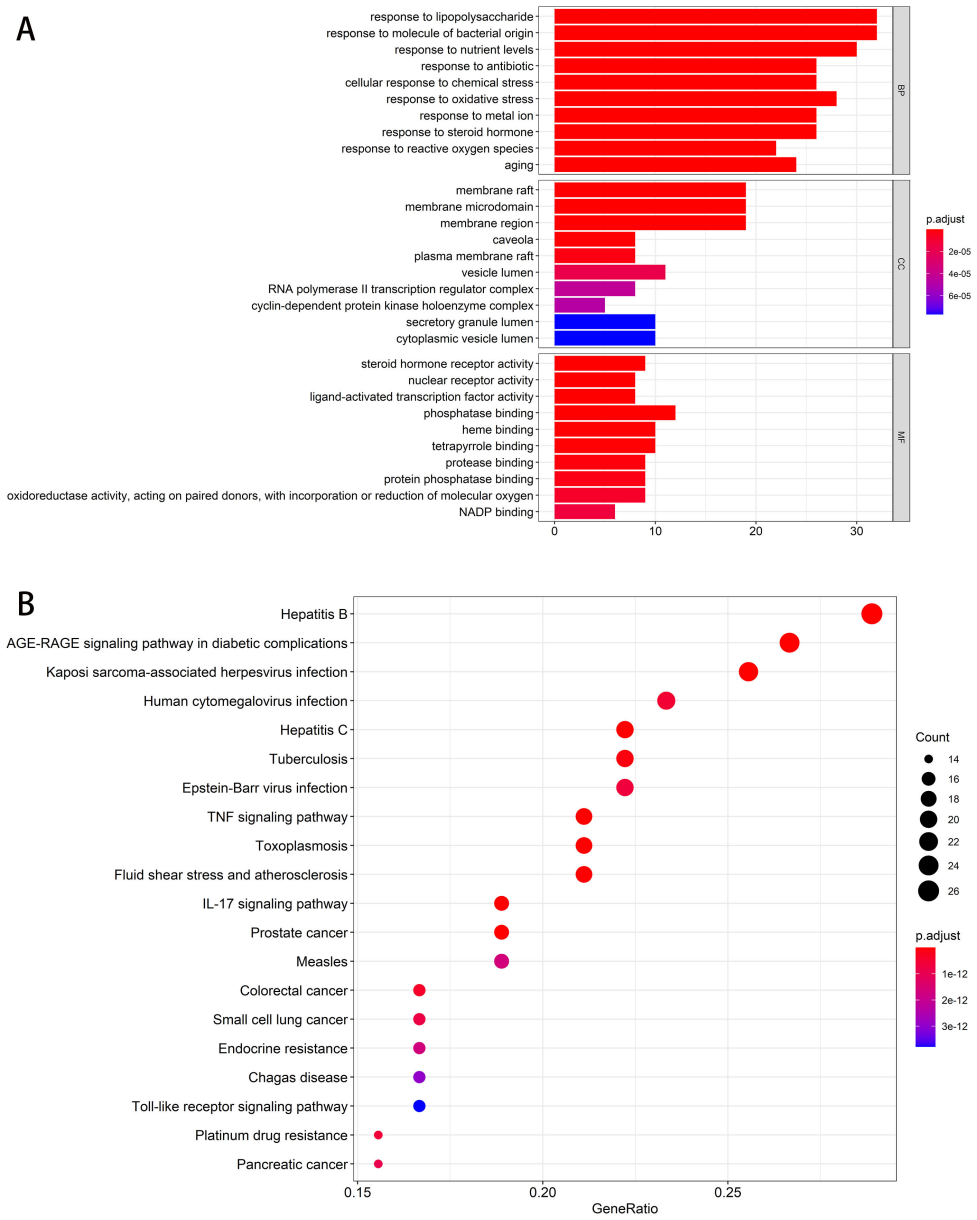
Enrichment analysis. **(A)** Bar plot for top 10 enriched GO terms of each sub-class. The length of each bar stands for enriched counts, and the color of each bar stands for adjusted p-value. The abscissa represents the enriched gene counts. **(B)** Dot plot for top 20 enriched KEGG pathway terms. The size of each node stands for enriched counts, and the color of each node stands for adjusted p-value. The abscissa represents the enriched gene ratio.

The results showed that with a post-adjusted *p*-value threshold of 0.05, 94 targets had enriched 1935 biological processes (BPs), 48 cellular components (CCs), and 131 molecular functions (MFs). The top 10 enriched GO terms from each category were directly shown in **Figure 2A**. The results showed that targets were enriched in BPs related to antibacterial and inflammation, including the response to lipopolysaccharide, response to molecule of bacterial origin, and response to oxidative stress. The CCs and MFs results indicated that most targets were localized in the membrane and related to steroid hormone receptor activity, nuclear receptor activity, ligand-activated transcription factor activity, and phosphatase binding. Meanwhile, the top 20 enriched KEGG pathways were visualized in **Figure 2B**. The results showed that majority targets are associated with TNF signaling pathway, IL-17 signaling pathway, and Toll-like receptor signaling pathway. These findings suggest the complex pathological process of diarrhea and the therapeutic potential of TXYF as a treatment for diarrhea.

#### 3.1.3 PPI network analysis and hub targets screening

**Figure 3:**
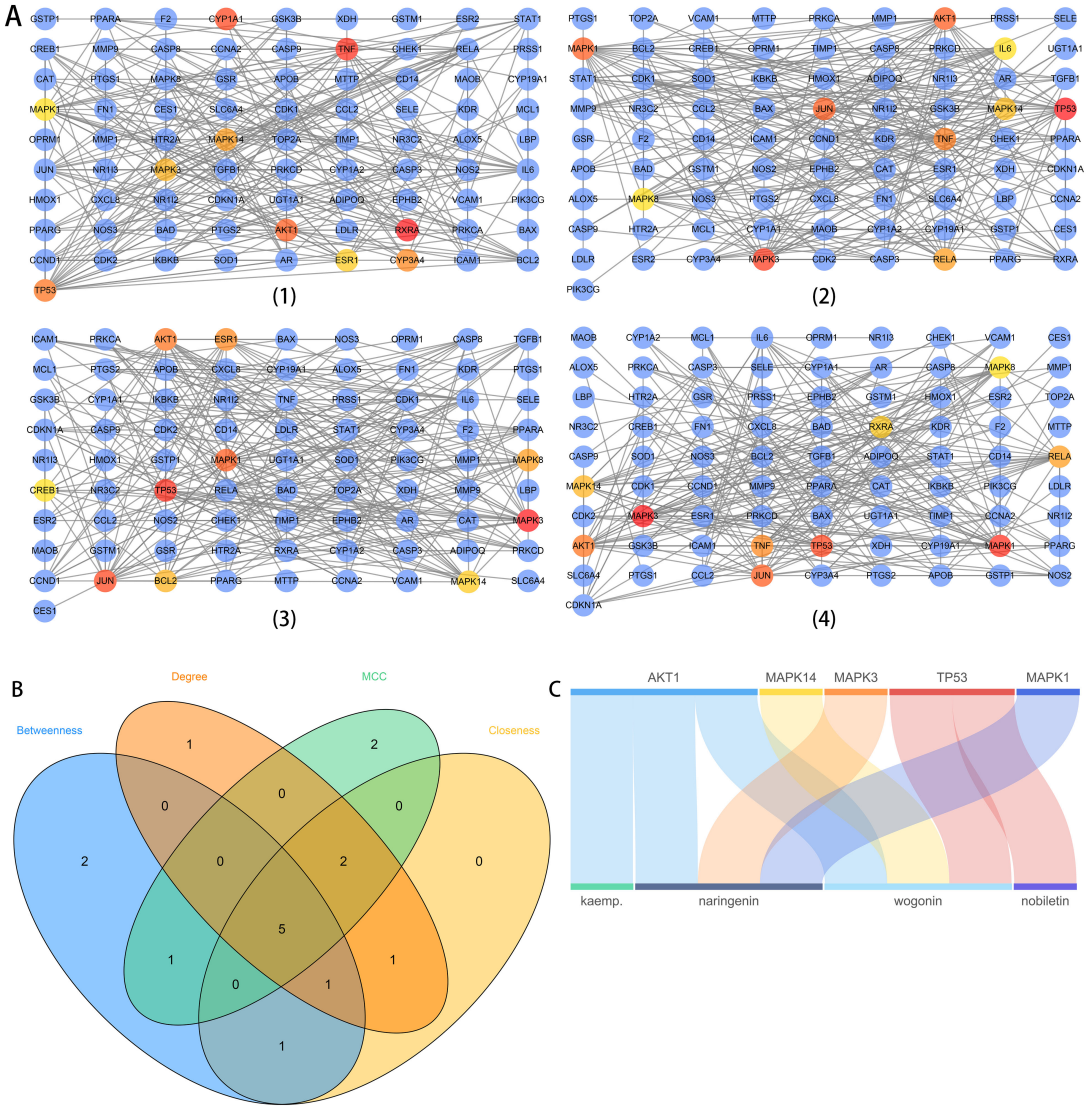
The PPI network analysis **(A)**, the Venn diagram of core hub targets **(B)**, and the Sankey diagram of core hub targets and corresponding compounds **(C)**. In **(A)**, (1) to (4) networks represent the analysis results based on four algorithms, Betweenness, Degree, MCC, and Closeness, respectively. The highlight red-to-yellow nodes are hub targets (top 10). The blue nodes are also protein targets with the highest confidence (0.9) from the PPI network analysis. In **(B)**, hub targets (top 10) identified from four algorithms were intersected to obtain the core hub targets for further analysis. In **(C)**, the relationships between core hub targets obtained from the Venn diagram and corresponding bioactive compounds were shown directly. The up blocks stand for the core hub targets, and the underneath blocks stand for compounds, as follows: kaempferol, naringenin, wogonin, and nobiletin.

To investigate the relationships among the 94 intersectant targets, we constructed a PPI network using the STRING database and visualized it with Cytoscape software. After removing disconnected nodes, a PPI network including 82 nodes and 282 interactions were constructed with the highest confidence score of 0.9 as the threshold. Hub targets were identified using four different algorithms, Betweenness, Degree, Maximal clique centrality (MCC), and Closeness, in the cytoHubba app (**Figure 3A**). Then, core hub targets of the PPI network were obtained by intersecting hub targets (top 10) identified from four algorithms (**Figure 3B**). Therefore, five targets (AKT1, MAPK14, MAPK3, TP53, and MAPK1) were identified as the core hub targets of the PPI network. Four bioactive compounds (kaempferol, naringin, wogonin, nobiletin) linked to five core hub targets were then selected in reverse. They were also the main compounds in TXYF. The relationships between core hub targets and corresponding bioactive compounds were shown directly using a Sankey diagram (**Figure 3C**).

### 3.2 TXYF administration altered the community diversity in intestinal mucosal microbiota in diarrhea mice

**Figure 4:**
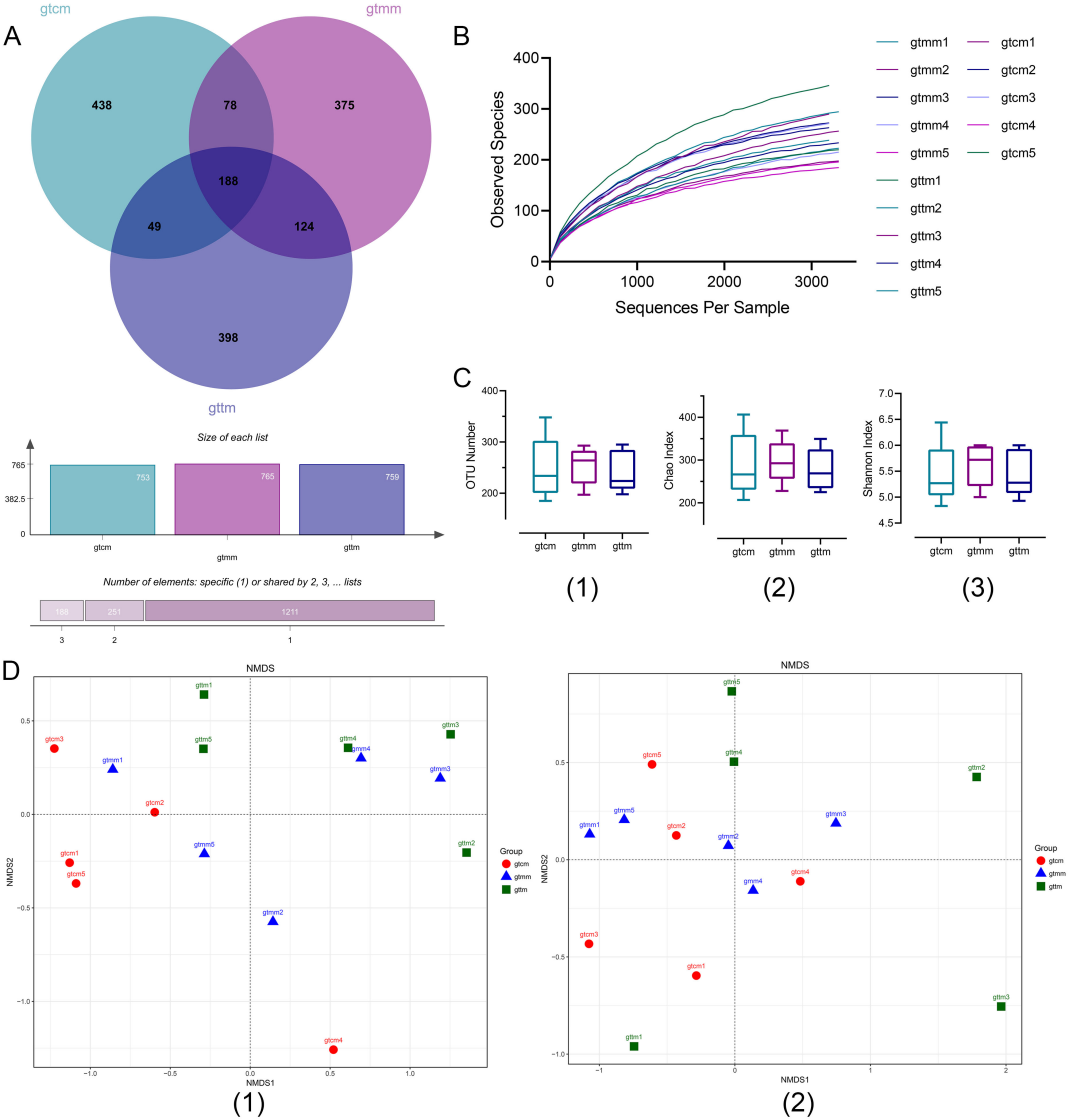
**(A)** Venn diagram of OTUs (at distance 0.03). **(B)** Rarefaction curve based on observed species. **(C)** Box plots of α-diversity index of OTUs number (1), Chao1 (2), and Simpson (3). **(D)** NMDS of OTU-based unweighted (1) and weighted (2) UniFrac distances. gtcm, the control group; gtmm, the placebo group; gttm, the TXYF group.

By high-throughput sequencing, 50005, 44322, and 42813 filtered high-quality sequences were obtained from samples of the control, placebo, and TXYF groups respectively, with the length concentrated at 1500 bp. A Venn diagram of OTU numbers showed that the gut microbiota community was changed with the treatment of TXYF (**Figure 4A**). The shared OTUs among the three groups were 188 (11.39%). The rarefaction curve demonstrated that the current sequencing depth was adequate to detect the microbial diversity in these community samples (**Figure 4B**).

Diversity analysis was performed at two levels – within the sample and across samples. Alpha diversity indicators, such as observed species, Chao1 and Shannon indexes, were applied to reflect richness and evenness of community within a single sample (**Figure 4C**). As the results suggested, the placebo group has the highest community richness among the three groups. Compared with the placebo group, community richness and diversity decreased in the TXYF group and reached the same level as the control group. However, there was no statistical differences among the three groups (*p* > 0.05). Nonmetric multidimensional scaling (NMDS) of OTU-based weighted and unweighted UniFrac distance was conducted to present the similarities and differences in community composition across different samples and groups (**Figure 4D**). NMDS highlighted a significant segregation of the gut microbiota according to the unweighted UniFrac distance (ANOSIM, R = 0.1724, *p* = 0.040; Adonis, R2 = 0.18382, *p* = 0.033), suggesting that TXYF administration was a potential factor in alterating community composition.

### 3.3 TXYF administration changed the community composition in intestinal mucosal microbiota in diarrhea mice

**Figure 5:**
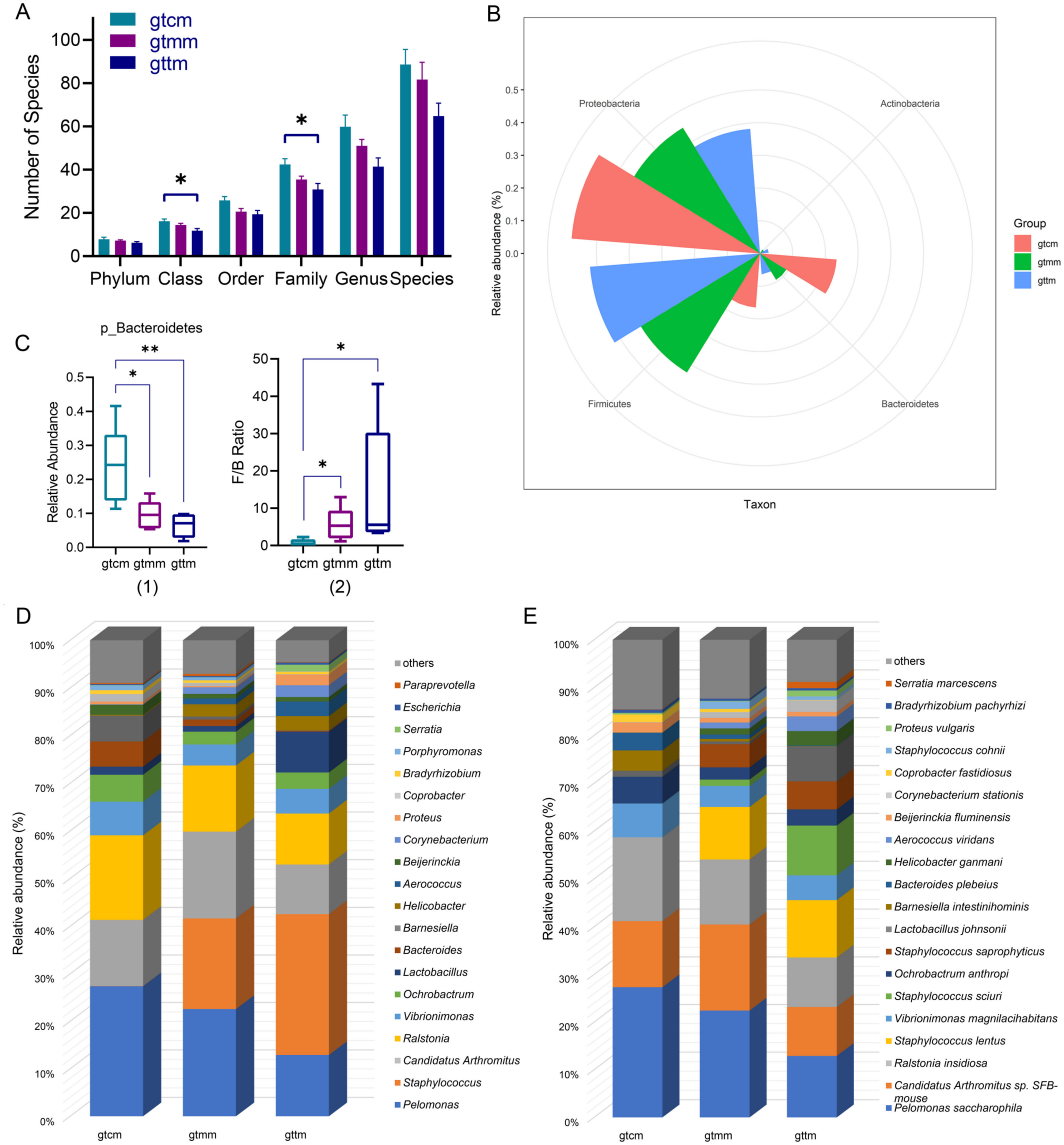
**(A)** Histogram of the number of microbial species of each sample at different taxonomic levels. **(B)** Radar graph of the relative abundance of dominant phylum in the intestinal mucosa. **(C)** Box plots of relative abundance of Bacteroidetes (1) and F/B ratio (2). **(D, E)** Stack diagram of relative abundance of genus **(D)** and species **(E)** in the intestinal mucosa. *p < 0.05, **p < 0.01, Kruskal-Wallis H test. gtcm, the control group; gtmm, the placebo group; gttm, the TXYF group.

To investigate whether there were representative taxa with differences, which may contribute to differences in diversity, we analyzed the community structure and composition of the three groups at the taxonomic level from phylum to species. OTUs were annotated to 17 phyla, 35 classes, 61 orders, 116 families, 199 genus, and 375 species. The proportion of unclassified OTUs was 4.06% (67/1650), suggesting that the full-length 16S sequencing technology has a high resolution in cluster analysis. In **Figure 5A**, the numbers of taxa at class and family levels in the TXYF group were significantly less than the control group (*p* < 0.05). In detail, compared with the placebo group, class Acidimicrobiia and 13 family taxa were newly colonized in the TXYF group, while seven class taxa and 21 family taxa were missing from the TXYF group.

Firmicutes (52.09%), Proteobacteria (38.21%), Bacteroidetes (6.41%), and Actinobacteria (2.69%), accounting for more than 98%, were the dominant communities in the intestinal mucosa of mice in the TXYF group (**Figure 5B**). Moreover, compared with the control group, the relative abundances of Bacteroidetes in both placebo and TXYF groups were significantly decreased, which resulted in a significant increase in the Firmicutes to Bacteroidetes (F/B) ratios in both groups (*p* < 0.05) (**Figure 5C**).

At the genus rank, dominant populations varied among groups (**Figure 5D**). Specifically, *Staphylococcus* (29.64%), *Pelomonas* (12.91%), *Ralstonia* (10.69%), *Candidatus Arthromitus* (10.41%), *Lactobacillus* (8.57%), and *Vibrionimonas* (5.18%), whose relative abundance was greater than 5%, were the six enriched genera in the TXYF group. They accounted for 77.4% of the total in the TXYF group. Only four genera had relative abundance greater than 5% in the placebo group, namely *Pelomonas*(22.59%), *Staphylococcus*(19.05%), *Candidatus Arthromitus*(18.21%), and *Ralstonia*(13.88%). They accounted for 73.73% of the total in the placebo group. The relative abundances of *Pelomonas*(27.39%), *Ralstonia*(17.74%), *Candidatus Arthromitus*(13.91%), *Vibrionimonas*(7.06%), *Ochrobactrum*(5.65%), *Barnesiella*(5.41%), and *Bacteroides*(5.28%)were greater than 5%, accounting for 82.44% of the total in the control group.

At the species level, enriched species with a relative abundance greater than 5% in the TXYF group were *Pelomonas saccharophila* (12.91% vs 22.44% in gtmm), *Staphylococcus lentus* (12% vs 10.98% in gtmm), *Staphylococcus sciuri* (10.4% vs 1.31% in gtmm), *Ralstonia insidiosa* (10.4% vs 13.59% in gtmm), *Candidatus Arthromitus* sp. *SFB-mouse* (10.27% vs 18.04% in gtmm), *Lactobacillus johnsonii* (7.3% vs 0.57% in gtmm), *Staphylococcus saprophyticus* (5.85% vs 4.84% in gtmm), *Vibrionimonas magnilacihabitans* (5.18% vs 4.41% in gtmm) (**Figure 5E**).

### 3.4 Microbial biomarkers associated with the administration of TXYF

**Figure 6:**
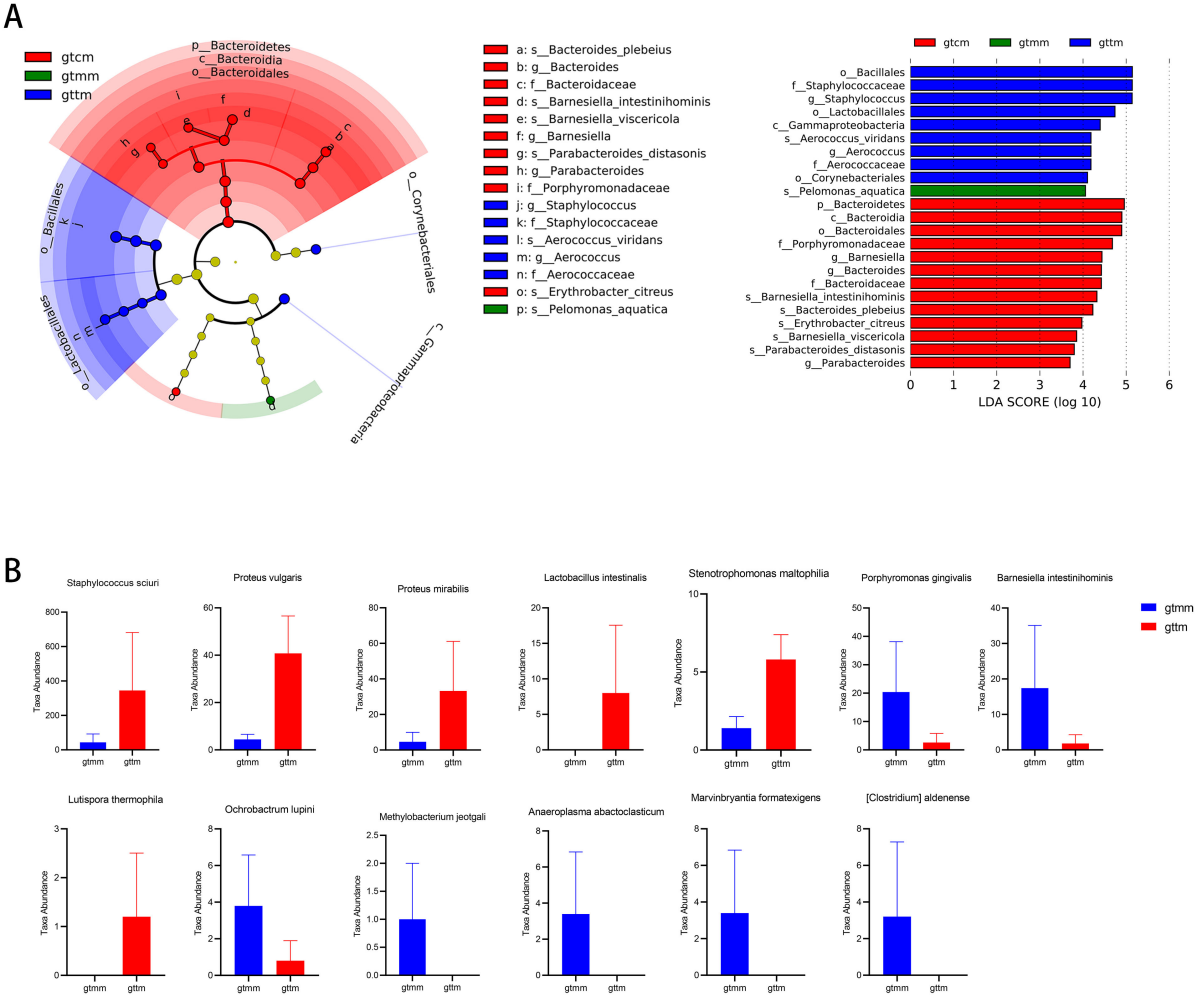
The potential biomarkers associated with the administration of TXYF. **(A)** Identified taxonomic features of significant differences across three groups by LEfSe (LDA score ≥ 4 and *p*-value ≤ 0.05). **(B)** Identified discriminative species between the TXYF (gttm) and placebo (gtmm) groups by Metastats analysis.

To further identify the biomarkers associated with TXYF administration, we used LEfSe analysis to analyze microbial abundances at taxonomic levels from phylum to species. A total of 23 specific microbial biomarkers were identified across the three groups with linear discriminant analysis (LDA) score greater than 4 and *p*-value less than 0.05 (**Figure 6A**). Taxa including Gammaproteobacteria, Bacillales, Lactobacillales, Corynebacteriales, Staphylococcaceae, Aerococcaceae, *Staphylococcus*, *Aerococcus*, and *Aerococcus_viridans* were significantly enriched in the TXYF group, which could be recognized as biomarkers associated with the treatment of TXYF.

In addition, the Metastats analysis based on the number of sequences (i.e., absolute abundance) was performed to compare the differences in composition at species level between the TXYF and placebo groups. As shown in **Figure 6B**, there were significant increases in six species, such as *Staphylococcus sciuri, Proteus vulgaris, Proteus mirabilis, Lactobacillus intestinalis, Stenotrophomonas maltophilia*, and *Lutispora thermophila*, and decreases in seven species, including *Porphyromonas gingivalis*, *Barnesiella intestinihominis*, *Ochrobactrum lupini*, *Methylobacterium jeotgali*, *Anaeroplasma abactoclasticum*, *Marvinbryantia formatexigens*, and *Clostridium aldenense*, in the TXYF group compared with the placebo group.

### 3.5 TXYF administration regulated the microbial function

**Figure 7:**
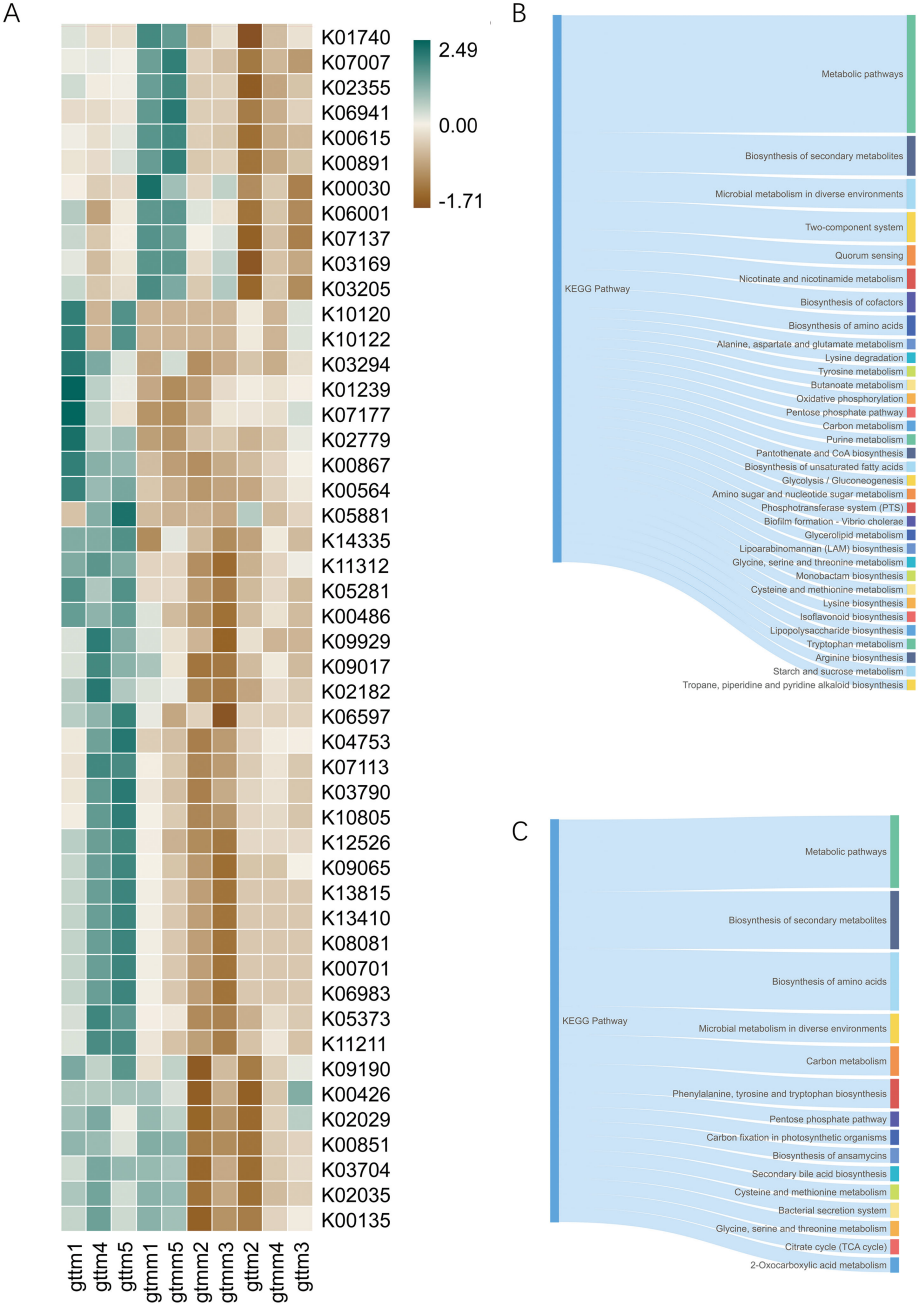
Function prediction based on the KEGG database by PICRUSt. **(A)** Heatmap for the relative abundance of discriminative KOs between the TXYF (gttm) and placebo (gtmm) groups. **(B)** The main KEGG pathway of the KOs with significantly increased expression in the TXYF group. **(C)** The main KEGG pathway of the KOs with significantly decreased expression in the TXYF group.

To predict the potential functions of microbial communities and assess the differences in microbial functions across groups, the BIOM file of OTU abundances was matched with the reference genomes of the KEGG database. The 16S sequencing dataset of 15 samples resulted in a functional dataset containing 5734 KOs. Further statistical analysis showed that 48 KOs were significantly altered between the TXYF and placebo groups (**Figure 7A**). Administration of TXYF significantly increased relative abundances of KOs in pathways such as two-component system, quorum sensing, biosynthesis, and metabolism of cofactor, butanoate, and tryptophan (**Figure 7B**). But in the meantime, TXYF reduced the relative abundance of KOs in pathways such as bacterial secretion system, secondary bile acid biosynthesis, and citrate cycle (**Figure 7C**).

## 4 Discussion

According to the theory of TCM, TXYF was widely used to treat diarrhea due to liver stagnation and spleen deficiency syndrome in clinical practices. A meta-analysis of randomized trials also indicated that TXYF as a treatment for IBS-D was more effective and safer in improving the clinical effectiveness rate and decreasing the adverse events than conventional medication such as probiotics, pinaverium bromide, trimebutine, and Oryzanol (Zhou et al., 2019b). The clinical efficacy of TXYF is positive, but the mechanism of TXYF relieving diarrhea is indefinite. *Folium senna* combined with restraint stress is an effective method of establishing the mice model of liver stagnation and spleen deficiency syndrome (Yuan et al., 2020; Zhang et al., 2020). In this study, we used network pharmacology to identify potential active compounds and targets for TXYF to alleviate diarrhea and focused on the effects of TXYF on the intestinal mucosal microbiota of diarrhea model mice using PacBio SMRT sequencing technology.

Network pharmacology was an efficient approach to elucidate the intrinsic multi-component, multi-target, and multi-pathway features of Chinese medicine prescriptions. In this study, we obtained 32 compounds, including kaempferol, wogonin, naringenin, nobiletin, paeoniflorin, and Atractylenolide IIIt as active fractions of TXYF. The intestinal epithelium barrier consists of a single layer of enterocytes and tight junctions (TJs). A leaky barrier may contribute to hyper-intestinal permeability, which will lead to intestinal dysfunction and systemic diseases (Camilleri et al., 2017). Malfunction of TJ can also trigger the development of infection, inflammation, obesity, and even autoimmune diseases (Wen et al., 2020). Chen et al (Chen et al., 2020). reported that kaempferol supplementation up-regulated the expression of tight junction proteins, butyrate receptors, and transporters in the intestinal mucosa in acute alcoholic liver injury mice. The beneficial action of kaempferol on LPS-induced inflammation and barrier dysfunction in a coculture model of intestinal epithelial cells and intestinal microvascular endothelial cells was associated with inhibiting the NF-κB signaling pathway activation (Bian et al., 2020). In addition, studies suggested that supplementations of TCM bioactive compounds such as wogonin, naringenin, nobiletin, and paeoniflorin could suppress inflammatory response and maintain the intestinal barrier function (Azuma et al., 2013; Wang et al., 2015; Wu et al., 2019; Wen et al., 2020). Atractylodes III was also considered to have an inhibitory effect on mRNA and protein expressions of pro-inflammatory (IL-1β, TNF-α, IL-6) and anti-inflammatory cytokines (Zhou et al., 2019a).

Furthermore, the anti-inflammatory effect of TCM bioactive compounds was related to gut microbiota and its metabolites. It has been reported that the anti-arthritis activities of kaempferol were linked to functions of reversion the perturbation of metabolites involved in energy production and the tryptophan, fatty acid, and secondary bile acid metabolisms in the gut contents (Aa et al., 2020). Paeoniflorin has active impacts in decreasing the serum contents of corticosterone, corticotropin-releasing hormone, and adrenocorticotropic hormone and increasing serotonin and 5-Hydroxyindoleacetic acid in the prefrontal cortex and hippocampus in post-traumatic stress disorder mice (Qiu et al., 2018). Moreover, the anti-inflammatory and immunomodulatory actions of paeoniflorin were microbiota-dependent and related to indole-3-lactate and epithelial autophagy (Fan et al., 2020). Therefore, we speculate that the bioactive compounds of TXYF may interact with gut microbiota after oral administration and play roles in alleviating diarrhea by maintaining the intestinal epithelial barrier and regulating intestinal microbes and their metabolites.

GO and KEGG pathway enrichment analysis further verified the above inference. The effect of TXYF in alleviating diarrhea may be related to multiple BPs and pathways, such as the inflammatory responses and the immune systems, which are mediated by the TNF signaling pathway, the IL-17 signaling pathway, and the Toll-like receptor (TLR) signaling pathway. Specifically, TNF signaling has an active impact on maintaining epithelial cell tight junctions and intestinal barriers. It was stated that loss of intestinal barrier function mediated by TNF signaling was highly relevant to the inflammatory pathophysiology observed in Crohn’s disease and celiac disease (Kolodziej et al., 2011). TLR signaling pathway was proven to play an essential role in maintaining tolerance to commensal microbiota and regulating specific immune responses against pathogens, which is vital for the homeostasis of the intestine and activation of innate immunity (Takeda and Akira, 2004). For example, Yi et al (Yi et al., 2021). found that dietary anethole supplementation can alleviate intestinal barrier disruption and intestinal inflammation induced by enterotoxigenic *Escherichia coli* through modification of TLR signaling and intestinal microbiota. However, further studies are needed to confirm the exact role of these pathways in the process of TXYF relieving diarrhea.

Clear evidence from full-length 16S rRNA gene sequencing indicates that TXYF has a direct effect on the intestinal mucosal microbiota of diarrhea model mice. From a macro perspective, although no significant difference was observed in bacterial richness and evenness across groups according to alpha diversity analysis, beta diversity analysis based on unweighted UniFrac distance suggested a statistical difference in microbial composition among the three groups. We also found that the Chao1 and Simpson indices of the TXYF group were in the middle level of the three groups. These findings indicate that *Folium senna* combined with restraint stress might perturb the biodiversity in the intestinal mucosa by increasing bacterial richness, which is consistent with early studies (Zhang et al., 2020, 2021). TXYF might be a driving force in regulating the intestinal mucosal microbiota of diarrhea mice to a healthy state.

Then, we further concentrated on the changes in specific taxa related to the administration of TXYF. We recognized some positive taxa that were associated with the protective effects of TXYF. In this study, the relative abundance of *Lactobacillus* increased significantly in the TXYF group. *Lactobacillus*, a commensal inhabitant of animal and human gastrointestinal tracts, is considered friendly bacteria with a wide range of applications from probiotics to therapeutic bacteria in both humans and animals (O’Flaherty et al., 2020). In our study, *Lactobacillus johnsonii* and *Lactobacillus acidophilus*, which are important members of the *Lactobacillus acidophilus* complex (LAC) (Ramachandran et al., 2013), were enriched with the administration of TXYF. *L. johnsonii* completely lacked genes encoding biosynthetic pathways to produce essential nutrients, such as amino acids, purine nucleotides, and cofactors. Thus, it has high nutritional requirements for the intestinal environment, and its development and function depend on the host or other intestinal microorganisms to maintain (Pridmore et al., 2004). Co-occurrence network analysis showed that *L. johnsonii* was positively correlated with *L. intestinalis*, suggesting a mutualism relationship between *L. johnsonii* and *L. intestinalis*. It has been established that *L. johnsonii* L531 could alleviate *Salmonella* infantis-associated enteritis and promote intestinal secretory IgA production via regulation of NLRC4 and NLRP3 inflammasomes, NF-κB signaling pathway activation and suppression of mitochondrial damage (Xia et al., 2020). Oral pre-administration of *L. acidophilus* played a protective role in dextran sodium sulfate-induced colitis by targeting the intestinal TJ barrier that involves the TLR-2 receptor complex (Al-Sadi et al., 2021). Based on the effect of inducing the expression of µ-opioid and cannabinoid receptors in intestinal epithelial cells and mediating analgesic functions in the gut, L. acidophilus was recognized as a potential therapeutic approach for abdominal pain and irritable bowel syndrome (Rousseaux et al., 2007). However, further studies are warranted to investigate the exact beneficial mechanism of *L. johnsonii* and *L. acidophilus* in diarrhea mice with liver stagnation and spleen deficiency syndrome.

## 5 Conclusion

In this study, network pharmacology and full-length 16S rRNA gene sequencing were used to investigate the active compounds and targets of TXYF in alleviating diarrhea and the effect of TXYF on the intestinal mucosal microbiota of diarrhea model mice. Kaempferol, wogonin, naringenin, and nobiletin are compounds associated with the efficacy of TXYF in relieving diarrhea. TXYF may alleviate diarrhea via multiple BPs and pathways, including TNF signaling pathway, IL-17 signaling pathway, and Toll-like receptor signaling pathway, which are involved in TCM-gut microbiota-host interactions. TXYF was directly verified to remodel the diversity and composition of the intestinal mucosal microbiota in mice with diarrhea associated with live stagnation and spleen deficiency syndrome. *Lactobacillus*, primarily *Lactobacillus johnsonii*, was enriched by the administration of TXYF and were identified as being involved in the therapeutic role of TXYF in alleviating diarrhea. In conclusion, oral administration of TXYF may alleviate diarrhea through remodeling intestinal mucosal microbiota. Promoting the colonization of beneficial commensal bacteria in the intestinal mucosa through gut microbiota-host interactions may be a potential mechanism of TXYF in the treatment of diarrhea.

## Data Availability

The datasets presented in this study can be found in online repositories. The names of the repository/repositories and accession number(s) can be found below: https://www.ncbi.nlm.nih.gov/, PRJNA861669.

## Supplementary material

Supplementary Table 1 The information of obtained bioactive compounds of TXYF.
